# Modeling Crowded Environment in Molecular Simulations

**DOI:** 10.3389/fmolb.2019.00086

**Published:** 2019-09-11

**Authors:** Natalia Ostrowska, Michael Feig, Joanna Trylska

**Affiliations:** ^1^Centre of New Technologies, University of Warsaw, Warsaw, Poland; ^2^College of Inter-Faculty Individual Studies in Mathematics and Natural Sciences, University of Warsaw, Warsaw, Poland; ^3^Department of Biochemistry and Molecular Biology, Michigan State University, East Lansing, MI, United States

**Keywords:** protein dynamics, macromolecular crowding, coarse-grained models, molecular dynamics simulations, crowder models

## Abstract

Biomolecules perform their various functions in living cells, namely in an environment that is crowded by many macromolecules. Thus, simulating the dynamics and interactions of biomolecules should take into account not only water and ions but also other binding partners, metabolites, lipids and macromolecules found in cells. In the last decade, research on how to model macromolecular crowders around proteins in order to simulate their dynamics in models of cellular environments has gained a lot of attention. In this mini-review we focus on the models of crowding agents that have been used in computer modeling studies of proteins and peptides, especially via molecular dynamics simulations.

## 1. Introduction

Intracellular organelles—in addition to water molecules, ions, metabolites, and other small solutes—typically contain between 200 and 400 g/L of macromolecules such as proteins, nucleic acids, ribosomes, and lipids. These complex environments may impact biomolecular function *in vivo* via crowding and confinement. The most obvious consequence is reduced diffusion. However, crowder molecules may also influence macromolecular folding and stability, internal dynamics and the sampling of functionally relevant conformations, complex formation, ligand binding and product release, catalytic activity, and other events (Zhou et al., 2008; Rivas and Minton, 2016).

In the majority of simulation studies, the functional dynamics of a given biomolecule has been investigated one molecule at a time and in the presence of only water and ions. However, when biomolecules experience crowding, the available volume is decreased and interactions with other biomolecules are unavoidable. This influences their diffusion and association pathways. Experiments increasingly study the function and dynamics of biomolecules under crowded conditions (e.g., Kuznetsova et al., 2014; Cheng et al., 2018; Fonin et al., 2018; Maximova et al., 2019). Thus, it is necessary to account for crowded conditions in simulations as well. Indeed, in the last decade, the number of studies of biomolecular interactions that consider not only water and ions but also other binding partners, metabolites or crowders has increased.

Crowding has the most pronounced effects on proteins with intrinsically disordered fragments or those that undergo significant conformational transitions as part of their function, for example during ligand binding. This applies to a vast majority of proteins. Therefore, it is time to establish standard protocols for how to include crowded environments in molecular simulations. This mini-review offers a brief guide through viable candidates. There are many reviews about the simulations of crowding, but we specifically focus on the crowder models used in molecular dynamics (MD) simulations. Other reviews cover the overall effects of crowding (Zhou et al., 2008; Christiansen et al., 2013), models of cellular environments at different scales (Feig and Sugita, 2013; Im et al., 2016; Feig et al., 2017), diffusion (Długosz and Trylska, 2011), and protein-protein interactions (Bhattacharya et al., 2013) in crowded environments.

## 2. Reduced Models of Crowders

**Figure 1:**
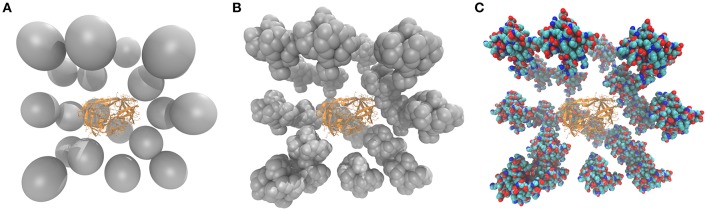
The HIV-1 protease surrounded by spherical **(A)**, CG **(B)**, and all-atom **(C)** crowders. In **(B,C)** the subdomain of a chicken villin was used as a crowding protein. The CG villin model **(B)** was built by representing each Cα atom as a 3 Å sphere.

A simple model for mimicking the excluded volume effect is geometric confinement where the physical volume available to a molecule is constrained. Typically, a spherical potential is applied, which restricts the conformational and diffusional freedom of the molecule similar to what explicit crowders would do. A similar approach is to penalize increased solvent-accessible surface areas (Tanizaki et al., 2008). More sophisticated models, which account for both the volume restraint and the presence of mobile crowders, include randomly placing explicit crowders around the molecule and applying appropriate boundary conditions. The most common model is to simulate a single molecule, in most cases a protein represented in a coarse-grained (CG) manner, surrounded by spherical crowders. By default, such a crowder is modeled as a single pseudo-atom with an enlarged radius to match the volume of a crowding agent of interest (Figure 1A). Typical crowder particle radii vary between 10 and 50 Å, with an average of 25 Å. Such sizes are appropriate to represent folded proteins or crowding polymers like Ficoll.

### 2.1. Single-Particle Spherical Crowders

Spherical crowders provide the excluded volume effect without requiring any specific interactions with the biomolecule. Therefore, crowder-molecule and crowder-crowder interactions are limited to van der Waals interactions via the Lennard-Jones potential and often only the repulsive part of the potential is considered (Minh et al., 2006; Kim et al., 2010). As the number of atoms in such systems is restricted to a minimum, the simulations become relatively fast, especially when an implicit water model is used. As a fast and simple solution, spherical crowders became popular and have been adopted in many types of simulations. They are often used in Brownian dynamics (BD) simulations (Cheung et al., 2005; Minh et al., 2006; Stagg et al., 2007; Wieczorek and Zielenkiewicz, 2008; Oh et al., 2014), but they can be also used in other methods such as MD (Kim et al., 2014; Miller et al., 2016) and Monte Carlo simulations (Kim et al., 2010).

Simulations with spherical crowders can be classified as a mixed-resolution approach because typically the crowders are represented as single particles, whereas biomolecules of interest are represented at higher levels, with at least one bead per residue. If the protein is represented with a CG model, some details about its behavior may be lost, such as its internal dynamics or specific aspects about interactions with the environment such as explicit water, other biomoleculs, or ligands. However, many questions can still be addressed with this simple approach such as the impact on diffusion (Ridgway et al., 2008), the stability of proteins (Cheung et al., 2005; Stagg et al., 2007) and protein complex formation (Kim et al., 2010, 2014; Latshaw et al., 2014) or inter-domain mobility (Minh et al., 2006). Some of these examples are briefly described below.

Single-particle crowders were shown to mildly stabilize some globular proteins, such as the native state of the WW domain (Cheung et al., 2005). The apoflavodoxin protein also favored more compact states at 25% vol. crowding (Stagg et al., 2007). A study on the HIV-1 protease (Figure 1A) showed that the frequency of opening of the protease flaps covering the active site is suppressed at high crowder fractions but low 5% vol. crowding was found to actually enhance the flap dynamics (Minh et al., 2006).

A simulation of amyloid aggregation suggested that crowding increases the rate of oligomer formation and fibril growth (Latshaw et al., 2014). These effects were found to depend on the size of the crowder particles, where smaller crowders enhanced the oligomerization rate to a greater extent. A similar enhancement was also seen in a simulation of antibody-antigen association under crowded conditions (Wieczorek and Zielenkiewicz, 2008).

The effect of crowding on the interactions between proteins forming complexes was also investigated. The binding free energy in two protein complexes (ubiquitin/UIM1 and cytochrome c/cytochrome c peroxidase) was shown to decrease in the presence of higher concentrations of repulsive crowders (Kim et al., 2010). Repulsive crowders also modestly stabilized the interactions in the pKID-KIX protein complex (Kim et al., 2014), but including an attractive term for protein-crowder interaction could destabilize the interaction in the protein complex.

Spherical crowders have also been used to study the impact of crowding on the conformational dynamics of intrinsically disordered proteins (IDP). In one study, the crowders were reported to induce compaction of disordered peptides (Miller et al., 2016). The compaction increased with decreasing radius of the crowders and with increasing volume fraction, but the effects also strongly depended on the peptide sequence.

### 2.2. Many-Particle Crowders

The resolution of the crowder particle can be increased by distributing a set of small pseudo-atoms on the surface of a sphere to form a bead shell (Elcock, 2003; Kurniawan et al., 2012). Such a model can match the higher resolution of a biomolecule of interest better and offer computational advantages as shorter non-bonded cutoffs can be used. Bead-shell crowders have been used in a BD simulation to calculate the free energy of the escape of a protein from the GroEL cage (Elcock, 2003) and later on in an MD simulation with explicit water molecules to observe the conformational changes of a short peptide (Kurniawan et al., 2012). In the latter work, crowding was found to facilitate folding of a β-hairpin by promoting compact structures and preventing unfolding of the intermediate conformations. Modeling crowders as CG proteins placed around a biomolecule represented with a CG model of similar resolution is also a computationally feasible option (Figure 1B). Such an approach was applied e.g., in BD simulations of ligands associating with HIV-1 protease in the presence of glutathione S-transferase P as a crowding agent (Kang et al., 2011).

Other variants of the spherical model, are dumbbell-shaped objects (Christiansen et al., 2010; Chen et al., 2012), where two spheres are linked by a harmonic bond, spherocylinders (O'Brien et al., 2011; Kang et al., 2015), and polymer chains (Nguemaha et al., 2018) with parameters adjusted to represent proteins, DNA or other polymers like polyethylene glycol (PEG). A good model of a cellular environment may require a mixture of spherical and cylindrical crowders and it has been found that such a mixture leads to different results than with crowders of only one type (Kang et al., 2015). In this work, the simulation of a DNA fragment revealed that the DNA conformation “swells” under crowded conditions and that crowders of mixed shape affected the conformation to a greater extent than each of the homomorphic crowders.

In a recent study (Zegarra et al., 2019), a set of crowders with different shapes was used to reproduce and explain an NMR experiment and show that the unfolded apoazurin protein becomes more extended upon addition of dextran crowders. In this work, spheres and spherocylinders of various lengths were used along with a CG protein model. The crowders that best reproduced the experimental results were elongated rod-like structures, interacting with the protein with repulsive and attractive potential terms.

Other studies have also confirmed that spherocylindrical crowders may induce different effects than spherical crowders. It was noted, that in the presence of spherical crowders, the compaction of a polymer increases with decreasing crowder radii, but the effects of spherocylindrical crowders are highly non-monotonical (Chen and Zhao, 2019). Spherocylinders were also shown to increase protein oligomer formation to a noticeably greater extent (O'Brien et al., 2011).

## 3. Capturing Atomistic Details

The most detailed information about the effects of a crowded environment can be obtained when the biomolecule and crowders are represented in atomistic detail (Figure 1C). In this case, not only the excluded volume effects can be explored but specific interactions between the molecule and crowders can be considered. As the level of realism increases, the question arises what kind of crowder molecules are best suited. The most realistic option would be to use a full model of a cytoplasm, with different proteins, nucleic acids, and metabolites (Yu et al., 2016). However, such an approach is computationally demanding and requires specific knowledge about the composition of specific cells. Other choices for all-atom crowders are small, well-studied proteins, like villin (Harada et al., 2013), protein G or trypsin inhibitor (Bille et al., 2019). Another aspect for choosing a specific crowder protein depends on what kinds of *in vitro* experiments a given simulation should be compared to.

All-atom simulations focused on the stability of protein native state in the presence of protein crowders represented in atomistic detail have suggested that crowding can promote local unfolding of the SOD1 protein (Bille et al., 2019) and destabilize the native state of villin (Harada et al., 2013). Destabilization of a pyruvate dehydrogenase subunit was also observed in a simulation of a model cytoplasm fragment (Yu et al., 2016) and was attributed to protein-protein interactions with crowder proteins. These observations are in contrast to previous studies using CG crowders, focused on the excluded volume effect, that tended to emphasize a stabilizing effect on native protein structures. This suggests that a full account of crowding effects cannot neglect the specific nature of protein-crowder interactions.

The advantages of using both all-atom and CG crowders can be combined by using a multi-scale approach. Such schemes allow for example to simulate a central molecule of interest in atomistic detail, while crowders are represented with a reduced CG model that still retains protein-like characteristics (O'Brien et al., 2011; Predeus et al., 2012; Bille et al., 2015). Such mixed resolution approaches allow simulations to be more efficient while still providing a detailed picture of protein behavior under crowded conditions. However, multi-scale approaches present challenges, e.g., with respect to how interactions between different levels of resolutions are treated.

One example for such a multi-scale approach, was the sampling of Trp-cage and melittin peptides (Predeus et al., 2012) in implicit solvent and in the presence of protein crowders represented with the PRIMO CG model. It was found that for both peptides, the addition of crowder molecules resulted in a more diverse conformational ensemble, with a larger share of non-native states.

In a multi-scale approach, crowders can be represented either as CG proteins or via simpler spherical molecules. Another study (Bille et al., 2015) investigating the Trp-cage conformational sampling compared an atomistic simulation with a mixed-resolution approach where an all-atom peptide was combined with spherical crowders. It was found that while the spherical crowders had almost no effect on the peptide conformations, rigid atomistic BPTI proteins used as crowders promoted non-native conformations and as a result stabilized the helical fragment. Again, this study points to the important role of non-specific peptide-crowder interactions.

A multi-scale approach was also used to study the formation of oligomers by peptides known to be amyloidogenic (O'Brien et al., 2011). In this study, the all-atom peptide model was mixed with crowders represented as spheres or spherocylinders. The authors compared the effects of different sizes, shapes, and volume fractions of the crowders. The crowders had a destabilizing effect on dimers formed by the peptides, but, surprisingly, trimers were stabilized. Moreover, it was reported that increasing crowder sizes reduced the crowding effect, while spherocylindrical crowders had a greater destabilizing effect than spherical crowders.

Apart from direct simulations, where crowders are explicitly present in the simulations, post-processing techniques have also been proposed. In this method, the protein in all-atom or CG representation and the crowders are simulated separately. The conformations obtained for the protein are then randomly placed in the snapshots of the crowder-containing trajectory and weighted based on the fraction of successful insertions. The post-processing method was applied to study the effects of crowding on protein dynamics (Qin et al., 2010), protein folding and binding stability (Qin and Zhou, 2009), and the conformational sampling of disordered proteins (Qin and Zhou, 2013).

The most significant challenge with running atomistic simulations of crowded systems, including explicit water, is the high demand for computer resources. Another issue is related to detailed balance between protein-protein and protein-water interactions. Modern force fields have been found to overestimate the interactions between proteins resulting in too much aggregation (Petrov and Zagrovic, 2014). One proposed solution for the CHARMM force field is to strengthen water-protein interactions by scaling the Lennard-Jones interactions (Nawrocki et al., 2017). This has led to better agreement with NMR experiments. However, irreversible aggregation artifacts are not to be confused with transient cluster formation that has been noticed in several many-protein simulations (Nawrocki et al., 2017; von Bülow et al., 2019) and is believed to be an accurate reflection of crowded solutions.

## 4. Discussion

Simulations of crowded environments can be performed at various levels of detail with respect to both the crowder and biomolecule. CG models of a biomolecule are often combined with a reduced crowder representation such as simple spheres. When biomolecules are represented in atomistic resolution, the range of models of the crowded environment becomes wider, ranging from spherical crowders and CG proteins in multi-scale approaches to all-atom protein crowders.

The crowders of choice for many researchers are spherical repulsive particles. They can be used in a variety of simulation methods and have been tested extensively. However, recent studies have shown that such models are likely oversimplified. The shape of the crowders and the way they interact can influence the effects that the crowders exert on the molecules (O'Brien et al., 2011; Kang et al., 2015; Chen and Zhao, 2019; Zegarra et al., 2019), including how crowding affects protein diffusion (Balbo et al., 2013).

Choosing a crowder model is a matter of finding a compromise between the allocated computational resources and simulation realism. Most detailed information about the impact of the crowded environment can be obtained if both the biomolecule and crowders are represented with atomistic details. Such models can provide insight into crowding effects well beyond the simple excluded volume effect. Including all-atom crowders may be especially vital to study peptides or IDPs since the interactions with the crowders can contribute significantly to the stabilization of their conformations other than those formed in bulk water or found in crystal structures.

The impact of crowding is a sum of often counteracting effects: the excluded volume effect and non-specific interactions of a biomolecule with the crowders. The importance of each component is not easy to predict as it may be case dependent (Rivas and Minton, 2016). With each level of reducing the representation of crowders, information about protein-crowder interactions is gradually lost, which is the main source of possible inaccuracies of simulations using CG crowders.

It has been shown that various sizes, concentrations, and shapes of CG crowders may differently influence the dynamics, interaction and diffusion of biomolecules. Therefore, the decision about the type of crowders is important and depends on the problem and questions that are being investigated, as well as the experiments with which simulations are being compared. Using atomistic representation is especially important while investigating the internal dynamics of biomolecules to compare with high-resolution structural experiments such as NMR spectroscopy. On the other hand, lower-resolution crowders may be sufficient to compare with experiments that emphasize non-biological space-filling crowders where the exact molecular nature is not as critical. One promising approach to account for both interaction details and reduce computational costs involves the use of mixtures of crowders with diverse properties. This may include crowders of different shapes, like spherical and spherocylindrical crowders (Kang et al., 2015), or a mixture of protein crowders such as the streptococcal protein G and the chicken villin head piece (Harada et al., 2013).

Finally, another question to consider while designing simulations of crowded environments is whether solvent needs to be accounted for explicitly. Explicit water typically requires fully atomistic simulations or high-level CG models although explicit water has also been combined with bead-shell crowders (Latshaw et al., 2014). On the other hand, if implicit solvent models are applied, questions about how to account for hydrodynamic effects arise (Ando and Skolnick, 2010; Długosz et al., 2011).

According to our benchmarks, surrounding a 236 amino-acid protein (in implicit solvent) with CG crowders has little to no effect on the simulation time. However, adding all-atom crowders (216 atom PEGs) to the same system slows down computations 3–5 times. For solvent treated explicitly, adding CG crowders can make the simulation faster because the crowders possess less atoms than water molecules that occupy similar volume. For example, 43-atom bead-shell crowders added to a protein-explicit solvent system (at 20% vol.) speed up the simulation by 20% as compared to simulations without crowders.

## Author Contributions

NO performed literature search. All authors wrote the manuscript, read, and approved the submitted version.

### Conflict of Interest Statement

The authors declare that the research was conducted in the absence of any commercial or financial relationships that could be construed as a potential conflict of interest.
